# Leaky gut, circulating immune complexes, arthralgia, and arthritis in IBD: coincidence or inevitability?

**DOI:** 10.3389/fimmu.2024.1347901

**Published:** 2024-03-20

**Authors:** Xi-ya Jin, Dan-dan Li, Wei Quan, Yang Chao, Bin Zhang

**Affiliations:** ^1^ Department of Gastroenterology, China-Japan Union Hospital of Jilin University, Changchun, China; ^2^ Department of Neurology, China-Japan Union Hospital of Jilin University, Changchun, China

**Keywords:** antigen-antibody complex, arthritis, leaky gut, inflammatory bowel diseases, complement, mitochondrial dysfunction

## Abstract

Most host-microbiota interactions occur within the intestinal barrier, which is essential for separating the intestinal epithelium from toxins, microorganisms, and antigens in the gut lumen. Gut inflammation allows pathogenic bacteria to enter the blood stream, forming immune complexes which may deposit on organs. Despite increased circulating immune complexes (CICs) in patients with inflammatory bowel disease (IBD) and discussions among IBD experts regarding their potential pathogenic role in extra-intestinal manifestations, this phenomenon is overlooked because definitive evidence demonstrating CIC-induced extra-intestinal manifestations in IBD animal models is lacking. However, clinical observations of elevated CICs in newly diagnosed, untreated patients with IBD have reignited research into their potential pathogenic implications. Musculoskeletal symptoms are the most prevalent extra-intestinal IBD manifestations. CICs are pivotal in various arthritis forms, including reactive, rheumatoid, and Lyme arthritis and systemic lupus erythematosus. Research indicates that intestinal barrier restoration during the pre-phase of arthritis could inhibit arthritis development. In the absence of animal models supporting extra-intestinal IBD manifestations, this paper aims to comprehensively explore the relationship between CICs and arthritis onset via a multifaceted analysis to offer a fresh perspective for further investigation and provide novel insights into the interplay between CICs and arthritis development in IBD.

## 1 Introduction

Inflammatory bowel disease (IBD) is a systemic condition that impacts the gastrointestinal tract; in addition, it affects various organs outside the digestive system in many patients (1). Extra-intestinal manifestations (EIMs) may be characterized as “inflammatory conditions occurring outside the digestive tract in individuals with IBD, either due to immune responses extending from the gut or as independent inflammatory events sharing genetic or environmental links with IBD” (1). These EIMs exert a significant influence on individuals with IBD, with reported prevalence rates extending to a maximum of 50% (2). Among these, musculoskeletal symptoms stand out as the most prevalent EIMs in IBD, impacting as much as 40% of patients (3). However, their pathogenic mechanisms remain unclear.

Recently, numerous reports have highlighted autoimmune diseases as being associated with gut microbiota dysbiosis (4). Supporting evidence exists for gut dysbiosis as an environmental trigger for arthritis in both animals and humans. Despite extensive research on gut microbiota composition, the direct cause of gut dysbiosis and its consequences in arthritis onset remain unclear. Gut microbiota dysbiosis compromises intestinal barrier function, elevating permeability and facilitating the entry of microbes, viruses, and pathogenic antigens into visceral organs (5). Patients with IBD showed significantly higher food-specific IgG antibody levels than healthy controls (6); autoantibodies reactive to colonic proteins (7) and anti-microbial antibodies have also been identified (8). In individuals with IBD, serum complement component 3 (C3) concentrations were elevated compared with those in healthy volunteers (9). Concurrently, immune complex-mediated inflammation has been suggested to influence certain extra-intestinal immune reactions associated with IBD (10). However, there is a lack of relevant research providing conclusive evidence demonstrating the initiation of extraintestinal manifestations (EIMs) by immune complexes in animal models of inflammatory bowel disease (IBD). In various types of arthritis, including rheumatoid and reactive arthritis (ReA), the significance of circulating immune complexes (CICs) has been emphasized. One potential mechanism underlying the exacerbation of arthritic conditions could be the disruption of the intestinal barrier.

To elucidate the pathogenic relevance of CICs in joint inflammation associated with IBD, this review comprehensively explores various dimensions of this subject. This review aspires to stimulate researchers to refocus their inquiries on this mechanism, thereby fostering relevant investigations.

## 2 Musculoskeletal EIMs

IBD, encompassing Crohn’s disease and ulcerative colitis, is an immune-mediated disorder marked by a chronic, relapsing-remitting course, significantly impacting the gastrointestinal tract (11). IBD affects the gastrointestinal tract and extra-intestinal organs of many patients. EIMs have the potential to impact diverse organ systems, emerging at any stage of IBD, possibly preceding gastrointestinal symptoms (1, 12, 13). In 1976, Greenstein et al. (14) summarized the characteristics and prevalence of EIMs in IBD by analyzing several patients with IBD.

In the rheumatologic context, as per the Assessment of Spondyloarthritis International Society criteria, musculoskeletal EIMs of IBD fall within the spectrum of spondyloarthritis (SpA) conditions (15). IBD-related musculoskeletal symptoms can impact both the axial and peripheral skeletal systems (16–18). The classification of peripheral musculoskeletal manifestations includes two types: Type I (oligoarticular), affecting fewer than five large joints and presenting as acute, asymmetrical, and migratory, and Type II, characterized by symmetrical arthritis impacting more than five facet joints, irrespective of intestinal disease activity (13, 19, 20).

Arthritis associated with IBD predominantly targets joints beyond the spinal column, including the knees, wrists, and ankles. Based on a comprehensive review of 69 studies, the median prevalence of axial SpA (axSpA) and peripheral SpA (pSpA) among patients with IBD was reported at 5 and 16%, respectively (21).

Diagnosis of IBD-associated arthritis relies primarily on clinical assessments, patient history, and exclusion of other arthritis forms (1, 22, 23). Treatment is primarily based on studies of SpA, and emphasizes addressing the underlying gut inflammation (13).

The mechanisms driving the development of musculoskeletal EIMs are believed to share similarities or commonalities with those underlying intestinal inflammation (24). Shared genetic factors between IBD and musculoskeletal EIMs suggest their contributory role. HLA-B27 positivity ranges from 25–78% in patients with IBD and ankylosing spondylitis (AS). Notably, approximately 60% of AS cases display symptomless gut inflammation, with a quarter progressing to manifest IBD (1). In a unique study, germ-free HLA-B27 transgenic rats exhibited no signs of gut or joint inflammation (25). Conversely, in non-germ-free transgenic rats, arthritis typically develops following episodes of diarrhea, closely mirroring the sequence observed in humans with enteritis-induced reactive arthritis (26). These findings suggest that bacterial exposure may serve as a pivotal prerequisite for the development of spondyloarthritis (SpA) in individuals genetically predisposed to inflammatory bowel disease (IBD).

Some mechanisms linking microbiota to extra-intestinal immune reactions have been explored. Dysbiosis may trigger the activation of immune cells in the intestines, leading to their migration to distant organs. Initial findings suggest heightened *Clostridiaceae* levels in individuals with IBD and arthritis (27), although this link appears moderately weak. Due to the compromised intestinal barrier, microbiota elements, such as lipopolysaccharides, bacterial antigens, or metabolites, may translocate from the gut to extra-intestinal locations, potentially inciting systemic inflammatory reactions (24).

## 3 The leaky intestinal barrier and formation of immune complexes in IBD

The intestinal barrier acts as a fundamental defense mechanism, shielding the host against potential microbial threats and averting immune responses (28). An array of diverse exogenous substances, including microorganisms, toxins, and antigens, inhabit the gut lumen. When the intestinal barrier lacks integrity or proper function, these substances infiltrate the tissues beneath the epithelial lining, diffusing into the bloodstream and lymphatic systems, disturbing tissue equilibrium (5, 28). The intestinal barrier, comprising physical, biochemical, and immunological components, plays a critical role. Our focus lies in understanding the immunological aspect, particularly highlighting the distinctive attributes of the B cell system in the context of IBD.

The gut immune system, the body’s largest and most intricate immune compartment (29), comprises two primary domains of B cell lineages. These encompass the organized B cell clusters residing in the gut-associated lymphoid tissue (GALT) and the diffuse lymphoid tissues spanning the extensive lamina propria of the small and large intestines (30). GALT serves as the initiation site for intestinal immune responses (31), housing various CD20-expressing B cell subtypes (32). Conversely, within the lamina propria, B lineage cells, mostly lacking CD20 expression, are believed to function as CD19+ plasma cell precursors (33). In the context of IBD, disrupted humoral immunity, marked by lymphoplasmacytic infiltrates—a recognized pathological hallmark—has been historically noted (34). IBD often prompts an increased formation of lymphoid aggregates harboring B cells and actively dividing T cells within inflamed mucosal tissues (35). These observations underscore the significant alterations in the gut’s immune landscape in IBD pathogenesis. Several studies have revealed increased microbiota-reactive IgG within the inflamed mucosa in IBD (36, 37); these antibodies circulate within the body and interact with bacteria associated with the mucosa (36). Serological investigations among individuals with IBD have highlighted noteworthy findings. For example, elevated levels of circulating anti-flagellin and anti-*Saccharomyces cerevisiae* antibodies were detected up to 5 years preceding the diagnosis of Crohn’s disease (38, 39). Moreover, a notable increase in fecal bacteria coated with IgG was noted in patients with IBD (40), and this measure strongly correlated with disease activity (41). Notably, autoantibodies have also been identified in IBD cases: anti-GM-CSF IgG is linked to severe complicated Crohn’s disease (6), whereas anti-TM5 IgG1 autoantibodies are specifically reported in ulcerative colitis (7). These serological profiles signify potential predictive and diagnostic markers, shedding light on the disease progression in different subsets of IBD. Food-specific IgGs and IgAs were also detected in patients with IBD, suggesting their reduced likelihood of being linked to food intolerance, as opposed to food-specific IgEs (42). Higher concentrations of antibodies against food proteins in the serum may suggest increased nutrient passage across the intestinal wall while preserving their antigenicity.

Recent findings challenge the prevailing notion that increased paracellular trafficking of gluten peptides through disrupted tight junctions precedes the onset of celiac disease (CD). Instead, observations of smaller gaps in tight junction areas in patients with CD suggest a strengthened upper epithelial barrier, potentially defending against the absorption of luminal antigens (43). Another study indicates that despite the presence of dilated tight junctions in CD and ulcerative colitis (UC), the transport of antigens OVA and HRP primarily occurs through normal ultrastructure enterocytes via transcellular pathways in all samples. Additionally, there is a more pronounced presence of enterocytes with electron-lucent cytoplasm containing a significant concentration of antigens in both CD and UC compared to healthy mucosa (44). These findings suggest that the increased intestinal permeability observed in IBD may be largely due to enhanced transcellular transport, leading to the production of several antibodies, including immune complexes.

**Figure 1:**
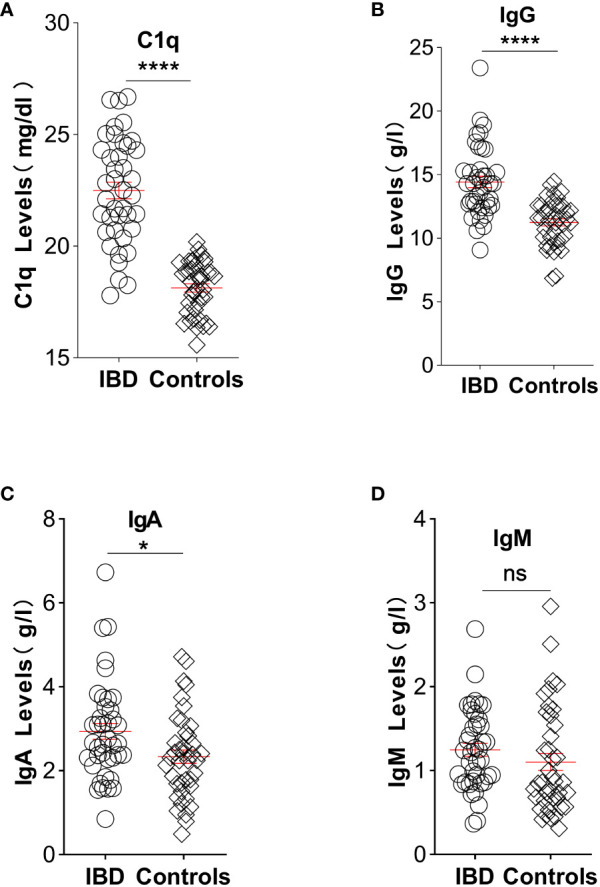
Illustration depicting the comparative analysis of C1q, IgG, IgA, and IgM levels between untreated IBD patients and individuals without chronic diseases: **(A)** Plasma levels of complement C1q significantly increased in patients with IBD (22.49, n=40) compared with controls (18.13, n=40). **(B)** Elevated IgG plasma levels were observed in IBD patients (14.44, n=40) compared with controls (11.25, n=40). **(C)** Patients with IBD exhibited higher concentrations of IgA in plasma (2.66, n=40) compared with controls (2.28, n=40). **(D)** Plasma levels of IgM showed no significant difference between IBD patients (1.23, n=40) and controls (0.86, n=40). Statistical significance is indicated as follows: ns indicates p > 0.05, * signifies p < 0.05, and **** denotes p < 0.0001 vs. the control group. (Unpaired t test with Welch’s correction of C1q and IgG; Mann Whitney test of IgA and IgM). Laboratory test results were collected from medical records of patients with IBD and age- and sex-matched healthy participants at the China-Japan Union Hospital of Jilin University between June 2021 and June 2022. Patients diagnosed with IBD had experienced symptoms for more than 6 months prior to hospital admission. In the experimental group, 47.5% of participants were male, whereas in the control group, the proportion was 35%. The mean ages of the experimental and control groups were 44.5 and 47.1 years, respectively. Exclusion criteria for patients included not receiving immunotherapy, absence of malignancy diagnosis, and normal body temperature. Healthy controls without any known diseases were enrolled and paired accordingly. The study adhered to the principles outlined in the Helsinki Declaration and the Rules of Good Clinical Practice. Approval was obtained from the Ethics Committee of the China-Japan Union Hospital of Jilin University, and the study was registered in the Chinese Clinical Trial Registry (No. 2023053016). All participants provided written informed consent before participation.

Elevated CIC levels have also been demonstrated in patients with IBD (29), and these levels correlate with disease activity and systemic presentation (45). Considering the presence of antigen-antibody responses in an inflamed gut, complement system activation would be expected. This system is crucial in maintaining intestinal immune homeostasis (46). Mice lacking key complement components exhibit increased intestinal inflammation in experimental colitis models, emphasizing the complement system’s importance in gut health (47). A murine gut cell line inherently expresses elevated levels of C3, toll-like receptor 2 (TLR2), and TLR4, with C3 activity notably rising upon exposure to lipopolysaccharides. Chronic colitis mouse models displayed escalated C3 levels across various intestinal tissues (48). Furthermore, a dextran sulfate sodium-induced experimental colitis model revealed heightened activation of GALT in the colon (35) alongside a significant accumulation of dense immune complexes on the intestinal epithelium (49). In a groundbreaking 1974 study by Ballard. et al., robust C3 and IgG staining were discovered on the lamina propria and basal membrane of intestinal epithelial cells in individuals with ulcerative colitis (50, 51). Notably, serum samples from actively affected patients with IBD exhibited elevated C3 levels compared with those from healthy controls (9). In our study, we gathered assay results pertaining to C1q, IgG, IgA, and IgM from untreated IBD patients within our department. These findings were juxtaposed with data obtained from individuals undergoing endoscopic gastrointestinal polyp resection, devoid of any chronic diseases. Notably, our analysis revealed significantly elevated levels of these indicators in untreated IBD patients in contrast to the control group, with the exception of IgM, as illustrated in **Figure 1**.

Immune complexes (ICs) play a vital role as biological mediators owing to their potential to induce tissue damage upon deposition in blood vessels or tissues. Among the three different types of ICs—small, intermediate, and large—intermediate-sized ICs typically cause the most damage as they become trapped in tissues or joints (52). Although the precise mechanism of IC deposition remains unclear, it is hypothesized that endothelial cells separate along the vessel wall owing to hydrostatic pressure and leakage, thus facilitating the entry of ICs (52). Once ICs become ensnared on the vessel wall, a series of inflammatory events ensues. If the corresponding class of antibodies is present within the IC, the complement system becomes activated, leading to mast cell degranulation and the recruitment of leukocytes to the site of IC deposition (53). Reactive leukocytes, particularly neutrophils, release lysosomal enzymes, such as elastase, collagenase, thrombin chain activator, cationic proteins, and kinin activators, thereby contributing to local tissue damage. This cascade reaction culminates in the destruction of blood vessels and tissues (54).

Despite various animal models demonstrating arthritis induced by immune complexes, their potential role in EIMs has been consistently overlooked. In the collagen-induced arthritis (CIA) mouse model, the onset of enteritis was observed prior to the development of arthritis, accompanied by increased intestinal permeability (55). Similarly, in CIA rats, although no significant inflammation or damage was observed in the colon, immunofluorescence staining revealed reduced Mucin 2 expression in colon tissue compared to that in colon tissues of controls (56). In the K/BxN spontaneous mouse model of arthritis, diminished ZO-1 expression was detected in the small intestinal (SI) and colonic epithelial cells of arthritic mice. Additionally, significant morphological changes, such as epithelial erosion and crypt elongation, were observed in the small intestine and colon of K/BxN mice with both early and established arthritis (57). These findings open avenues for a more comprehensive exploration of the impact of immune complexes on exacerbating extra-intestinal complications in IBD.

## 4 Gut dysbiosis and inflammatory arthritis

Inflammatory arthritis encompasses a range of rheumatic conditions characterized by synovial joint inflammation and systemic effects, with prevalent subtypes including rheumatoid arthritis (RA), psoriatic arthritis (PsA), and AS (58). Similar to other autoimmune diseases, many forms of inflammatory arthritis are associated with circulating autoantibodies (59–61). This type of arthritis, mediated by circulating autoantibodies, likely contributes significantly to joint inflammation in humans, as indicated by prominent synovial fluid complement fixation, the presence of immune complexes within synovial fluid neutrophils, and the identification of immune complexes in the joints of patients with RA (61–63).

Immune complexes, comprised of antibodies clustered around multivalent targets, primarily IgG, IgM, and occasionally IgA, induce an inflammatory response upon activation of the complement pathway. In joint tissues, immune complex accumulation primarily occurs through three pathways: direct deposition within joint tissues, local formation due to antibodies targeting intrinsic joint antigens, and interaction with antigens introduced into joints or generated within the joint, resulting in local immune complex formation. These pathways showcase diverse mechanisms underlying immune complex accumulation in joint tissues, contributing to elucidating the potential mechanisms driving joint inflammation across various conditions (64).

The human gut hosts an intricate ecosystem of microorganisms collectively termed the ‘gut microbiota,’ comprising approximately 10^13^ bacterial cells. This diverse microbial community, spanning over 250 species of viruses, fungi, bacteria, and archaea, exhibits dynamic changes throughout an individual’s lifespan. The symbiotic alliances among organisms in this community significantly influence various physiological and pathological processes, making their relationship profoundly mutualistic to human life (65). Gut dysbiosis, capable of disrupting the intestinal barrier integrity, potentially allows leakage of microbiota or their byproducts into gut tissues, possibly leading to their circulation within the venous or lymphatic systems (66).

The gut–joint axis signifies the correlation between gastrointestinal health and joint well-being, with gut dysbiosis being linked to the onset of several rheumatic conditions, including RA, axial SpA, PsA, and osteoarthritis (OA) (67–71). Numerous microbiome alterations in these conditions resemble those observed in chronic IBD, including reduced microbial diversity and decreased abundance of Firmicutes, known for their anti-inflammatory properties (71). Two systematic analyses of gut microbiota in untreated patients with early RA revealed an elevated prevalence of *Prevotella* species, notably *P. copri*, which was considerably less prevalent in the general population (72). Furthermore, studies indicated that *P. copri* exhibits pro-inflammatory effects in a murine colitis model (72). The latest Shotgun metagenomic sequencing studies have revealed that microbial taxonomic groups, functionalities, and even strains are shared between patients with arthritis and those with IBD. These alterations are largely consistent across RA, AS, and PsA, a finding previously unexplored in earlier research (73). Epithelial barrier dysfunction, observed in both murine models and human studies, has been noted in the preclinical stage of RA (74, 75). Li et al. utilized the Mendelian randomization method to unveil a significant association between PsA and IBD (76). Moreover, a cohort of patients with SpA exhibited clinical manifestations of IBD alongside latent gut inflammation (77–79). Recent insights underscore the pivotal involvement of gut microbiota as a primary mediator, amplifying immune complex deposition, complement activation, and macrophage infiltration. These mechanisms contribute significantly to the renal inflammation observed in systemic lupus erythematosus (SLE) (80).

## 5 Reactive arthritis

The World Health Organization and the International League of Associations for Rheumatology categorize joint relationships with infections into four groups (81). The first group includes pathogens found within joints that cause infectious arthritis originating from infections in other parts of the body. The second group includes post-infectious arthritis, where bacterial antigens are detected in the joint. The third group comprises ReA triggered by infections from the genitourinary or gastrointestinal systems, often undetectable in the joint. The fourth group involves microbial-induced inflammatory arthritis without the confirmed presence of the microbe, its products, or specific antigens within the joint.

In its early conception, ReA was characterized as non-purulent, developing subsequently into a gastrointestinal infection without direct bacterial infiltration into the joints (82). Kekomäki et al. (83) identified CICs in patients with intestinal infections and ReA. Patients with ReA exhibit the presence of microbial antigens and bacterial DNA and RNA within the synovial fluid or tissues of affected joints. Notably, these substances often indicate the persistent existence of metabolically active microorganisms (84–87). ReA commonly ensues following gastrointestinal infections caused by bacteria such as *Yersinia*, *Salmonella, Campylobacter*, and *Shigella*, with post-dysentery outbreaks, especially post-*Shigella*, being the most prevalent inciting events for this condition (88).

Recent reports on ReA encompass various rare causative microorganisms, including *Clostridium difficile* and *Escherichia coli* (89, 90). Several large human cohort studies have employed whole genome sequencing to reveal that *E. coli* (specifically adherent-invasive strains) and *Enterobacteriaceae* are typically elevated in individuals with IBD, reportedly enhancing the inflammatory response (91–93). Recurrence of *C. difficile* is common in IBD (94). Patients with IBD are notably more vulnerable to *C. difficile* infection (CDI), which can lead to elevated morbidity and mortality rates (95). Although whether IBD itself or disease activity is an independent risk factor for CDI remains unclear, additional predisposing and specific conditions have been proposed within this patient group (96, 97). One of the most prominent newly identified infectious agents linked to ReA is the SARS-CoV-2 virus (98). An immune complex, potentially comprising COVID-19 spike protein and associated antibodies, has been identified as a contributing factor to platelet activation and thrombosis in patients with COVID-19 (99). Nevertheless, no studies have established a definitive link between this complex and the underlying cause of ReA in COVID-19.

The clinical presentation of joint symptoms in both IBD and ReA is similar (**Table 1**). In the 20–30 age bracket, the prevalence of arthritis among patients with IBD is approximately 25%, while the strength of the HLA-B27 association in spondylitis-complicating IBDs ranges between 50 and 70% (1). ReA most commonly affects young adults in the 20–40 year age range, with 30–50% of patients carrying the HLA-B27 allele (100). The onset of ReA is often preceded by symptoms of the triggering infection, which may manifest as diarrhea in cases of gastrointestinal infections. In severe cases, this diarrhea may even cause an IBD such as Crohn’s disease (101). ReA presents with inflammatory back pain, arthritis, and extra-articular symptoms, mirroring the commonly observed manifestations in inflammatory bowel-associated arthritis (1, 102).

**Table 1:** A Comparison of arthritis in IBD and “classical” reactive arthritis.

	Arthritis in IBD	“Classical” reactive arthritis
Age	20–30 years predominantly	20–40 years predominantly
Gender	No significant difference	Male preponderance
Precipitating factor	Gut inflammation	Gut or urogenital infection
HLA-B27 association Strength	50–70%	30–50%
Phenotype	Axial arthropathy ankylosing spondyloarthritis -Isolated sacroiliitis -Inflammatory back pain peripheral arthritis - Oligoarticular asymmetric arthritis (involving preferentially large joints) - Polyarticular involvement (small joints of both hands)	Spondyloarthritis-like -Axial involvement -Lower limb predominant oligoarthritis
Chronicity	chronic	1/3rd become chronic (lasts beyond three months)
Management	Treatment of intestinal inflammation COX-2 inhibitors Corticosteroids (short term) Sulfasalazine (especially in ulcerative colitis) Methotrexate Anti-tumor necrosis factor	Treated as other spondyloarthritis cases (limited evidence base)
Extra-articular manifestations	Dactylitis	Dactylitis
	Enthesitis	Enthesitis
	Synovitis	Skin
	Uveitis	Uveitis
	Inflammatory bowel disease	Inflammatory bowel disease

## 6 Mitochondrial dysfunction and IBD

Within the colonic crypts, mitochondria maintain cellular energy gradients, which are crucial for effective cell differentiation and proliferation, and thus, pivotal in determining epithelial cell fate (103). Notably, five percent of the genetic susceptibility factors identified in human GWAS for IBD are associated with mitochondrial homeostasis (104). Moreover, research has revealed that spontaneous ileitis in Phb1^iΔIEC^ mice is attributed to mitochondrial dysfunction in all intestinal epithelial cells and early abnormalities in mitochondria in Paneth cells, with translational implications for the subset of patients with Crohn’s disease exhibiting Paneth cell defects (105). Research also indicates that p32 is the primary driver of mitochondrial oxidative phosphorylation, and goblet cell differentiation induction *in vitro* relies on p32-regulated mitochondrial function (103). Additionally, decreased p32 expression in UC is the fundamental cause of mitochondrial dysfunction and defective goblet cell maturation (103).

Notably, Ho et al. were the first to observe a significant increase in plasma mitochondrial DNA levels in patients with IBD and a dextran sodium sulfate-induced mouse model of intestinal inflammation (106). Moreover, N-formylated peptides derived from mitochondria were detected in both circulation and fecal samples (106). This indicates that mitochondrial damage-associated molecular patterns (DAMPs) are released pathologically within the inflamed mucosa of IBD. Given that mitochondria are endosymbionts originating from bacteria, their components are inherently immunogenic. Additionally, released mitochondrial DAMPs (MTDs), including its components, formyl peptides, and mitochondrial DNA, activate human neutrophils (PMNs) through formyl peptide receptor 1 and TLR9, promoting PMN Ca2+ influx and MAP kinase phosphorylation (107). Consequently, this triggers PMN migration and degranulation *in vivo*, ultimately leading to neutrophil-mediated organ damage (107). In recent years, a growing body of research has strongly suggested that mitochondrial dysfunction plays an important role in inflammatory arthritis (108). However, specific mechanisms require further elucidation. Therefore, it is imperative to confirm whether circulating mitochondrial components in patients with IBD form immune complexes as antigens and deposit in the joints, leading to inflammation.

## 7 Optimizing gut barrier for early arthritis management

The potential treatment approach for early and new-onset arthritis involves the restoration of intestinal barrier integrity. Emphasis is placed on the pivotal role of the intestinal barrier and microbial byproducts in various chronic illnesses. Elevated permeability within this barrier may serve as an initial trigger for a spectrum of diseases, encompassing gastrointestinal conditions or even exacerbating their progression. However, the precise causal relationship regarding this interplay remains to be conclusively established.

The permeability and barrier function of the intestinal epithelium relies on the regulation of intercellular tight junctions. Among various factors, intestinal permeability is controlled by the disengagement of protein ZO-1 from the tight junction protein complex, a process mediated by the zonula occludens toxin, also known as zonulin (109). Patients with RA have displayed significantly increased gut-permeability marker levels (55); arthritic mice have also been found to display increased intestinal permeability and inflammation (57). The serum zonulin levels in mice with collagen-induced arthritis also increased significantly well before the onset of arthritis (55). In recent adjuvant-induced arthritis rat models, increased intestinal permeability and gut inflammation precede the onset of arthritis (110). Treatment of arthritic mice with AT-1001, which prevents zonulin-mediated retraction of tight junctions, via oral gavage, has been found to prevent disruption of gut permeability, shown by significantly reduced FITC dextran uptake compared with that in untreated mice and significantly reduced joint swelling (57).

Short-chain fatty acids (SCFAs) - acetate, propionate, and butyrate - are produced through the anaerobic fermentation of dietary fiber by intestinal microbiota. They serve as essential nutrients for intestinal epithelial cells and support barrier function (111). Acetate is primarily predominant in the colon, while butyrate has garnered substantial attention in extensive research (112). A recent study has demonstrated that a decrease in butyrate-producing bacteria leads to lower levels of butyrate and decreased FFAR2/3 signaling, resulting in suppressed mucin formation, increased gut permeability, and inflammation (113). Dysbiosis in patients with CD is characterized by a decrease in butyrate-producing bacteria belonging to the order Clostridiales and the phylum Firmicutes (114). Additionally, a decrease in Roseburia, also within the order Clostridiales, contributes to the observed dysbiosis in patients with UC (115).

Although conclusive evidence does not strongly support disease-specific changes in gut microbes, consistent alterations have been observed in conditions such as RA, Sjögren’s syndrome, and SLE. These alterations involve a decline in anti-inflammatory butyrate-producing microbes like *Faecalibacterium* and an increase in pro-inflammatory microbes such as *Streptococcus* (67). In a recent study employing a quasi-paired cohort strategy, a decrease in butyrate-producing species and an enrichment of butyrate consumers was reported in patients with RA (116). Additionally, an underrepresentation of Lachnospiraceae, a key family of butyrate producers, has been linked to new-onset untreated RA, although its exact pathological significance remains unclear (117).

In patients with RA, a positive association has been noted between butyrate levels and the frequency of both total CD19+CD24hiCD38hiB cells and IL-10+B cells (118). Recent studies have unveiled that mild inflammatory cues governing the maturation of immature B cells into regulatory B cells originate within the GALT due to interactions between gut microbiota and the innate immune system (119). The strength of these inflammatory signals significantly influences the differentiation of B cells into either regulatory or mature B cells and the production of antibody-secreting plasma cells (120). Moreover, the use of butyrate to restore intestinal barrier integrity in the pre-arthritic phase has demonstrated inhibition of arthritis development (121).

**Figure 2:**
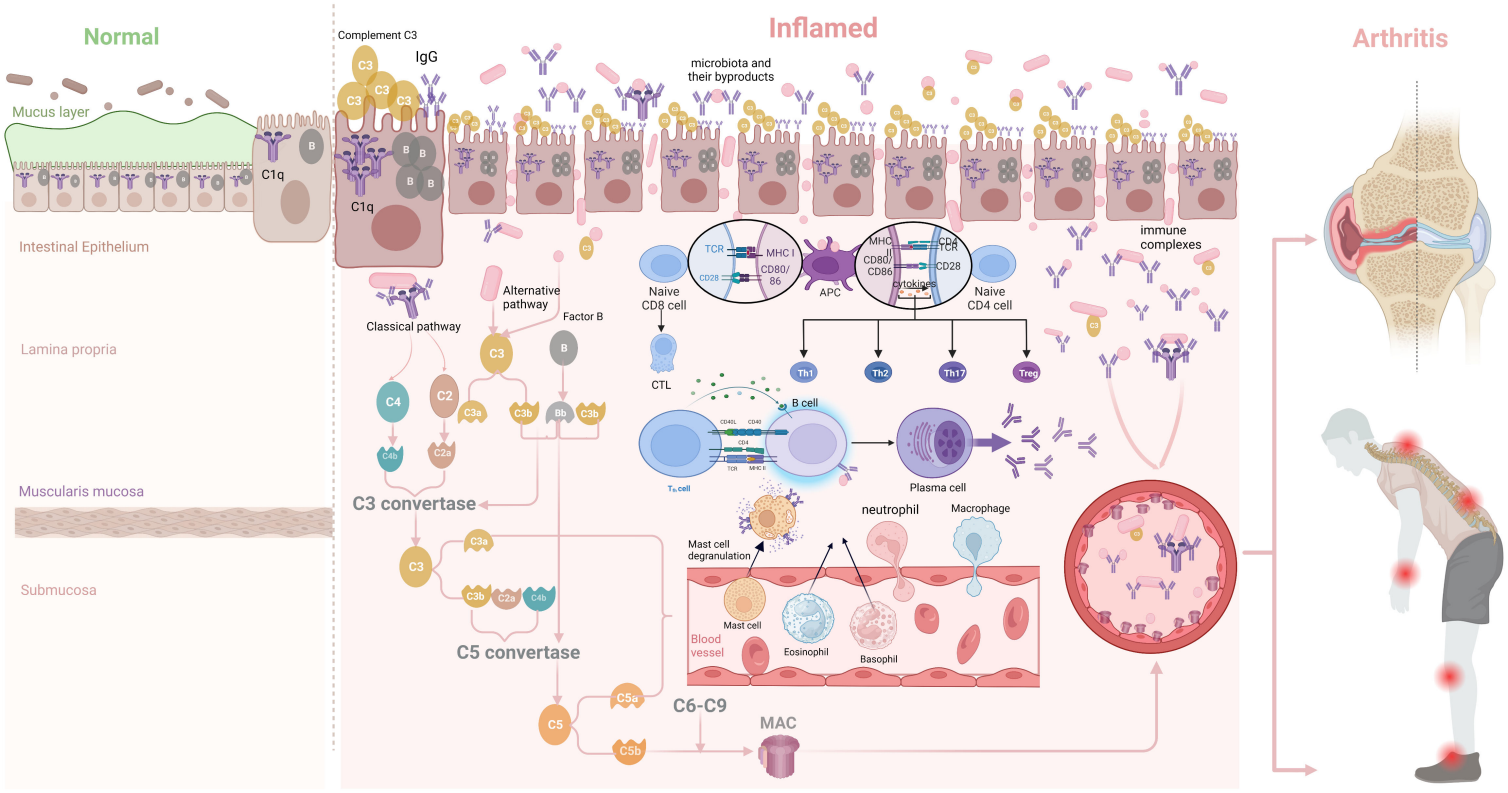
Inflammatory bowel disease leads to a compromised gut barrier, allowing the entry of microbiota and their products into gut tissues. This figure illustrates the interaction between intestinal epithelial cells, complement proteins (C1q, C3, factor B), IgG, antigen-presenting cells, T cells, and B cells. B cells capture microbial fragments, leading to their breakdown, while helper T cells activate B cells. This activation transforms B cells into plasma cells, producing antibodies. Additionally, the complement system forms membrane attack complexes (MAC) that adhere to affected cells and cause destruction. Some MAC structures attach to gut blood vessel walls, amplifying inflammation. The figure also highlights the potential for antibody-microbe complexes to travel through the bloodstream, potentially affecting joints. Created with BioRender.com.

In mice with established arthritis, compared with those in the early stages, bacterial 16S DNA accumulated in the mesenteric lymph nodes, paw-draining axillary lymph nodes, and spleen (57). Furthermore, arthritic K/BxN mice exhibited a significant increase in the frequency of LPAM-1+CCR9+CD45+ cells in various sites, including the spleen, Peyer’s patches, mesenteric lymph nodes, and paw-draining axillary lymph nodes, compared with those in control mice (naive nonobese diabetic mice) (57). These findings emphasize the association between arthritis development and compromised intestinal barrier integrity, suggesting the systemic spread of bacteria—a factor potentially contributing to arthritis pathogenesis. The decline in butyrate-producing bacteria in the intestine can be reasonably deduced to signify more than disruption of the intestinal barrier alone; instead, it likely triggers an increased generation of mature B cells and plasma cells, responsible for handling the abundance of antigens stemming from a leaky gut. This, in turn, results in the formation of immune complexes, which are likely key contributors to arthritis onset (**Figure 2**).

## 7 Conclusions and future directions

Some of the most significant progress in understanding the role of immune complexes in the musculoskeletal symptoms of IBD has emerged from unguided analysis of extensive datasets. Some of these findings “rediscover” concepts that have been long known and whose importance has been overlooked. For example, pioneers in rheumatology have long recognized that immune complexes represent a substantial pathway contributing to joint inflammation in humans. The potential for these complexes to induce inflammatory responses in joints has also been known. However, the focus in recent decades has predominantly been on T cells and the microbiota in the pathogenesis of IBD-related arthritis rather than on immune complexes. Recent research into the gut–joint axis has highlighted the direct connection between impaired intestinal barrier function, stemming from gut dysbiosis, and gut leakage, leading to arthritis. This once again emphasizes the pivotal role that immune complexes may play in this context. Nevertheless, the oversight of the impact of heightened CICs on EIMs in patients with IBD results from the lack of animal models demonstrating their involvement in initiating arthritis during enteritis. We are currently in an exciting era of unguided, in-depth, observational science, promising to not only prioritize established pathways but also to expand our knowledge.

## Data availability statement

The datasets presented in this study can be found in online repositories. The names of the repository/repositories and accession number(s) can be found in the article/**Supplementary Material**.

## Ethics statement

The studies involving humans were approved by Clinical Research Ethics Committee of China-Japan Union Hospital of Jilin University. The studies were conducted in accordance with the local legislation and institutional requirements. The participants provided their written informed consent to participate in this study. Written informed consent was obtained from the individual(s) for the publication of any potentially identifiable images or data included in this article.

## Author contributions

XJ: Writing – original draft. DL: Writing – review & editing, Investigation. WQ: Writing – review & editing, Supervision. YC: Writing – review & editing, Supervision. BZ: Writing – review & editing, Conceptualization.
